# The State of Climate Negotiations: a personal scientific commentary

**DOI:** 10.1186/1750-0680-8-5

**Published:** 2013-05-15

**Authors:** David E Archer

**Affiliations:** 1 Department of the Geophysical Sciences, 5734 Ellis Ave, Chicago, IL 60637, USA

## Abstract

Humanity seems unable to rein in its CO_2_ emissions, and yet the author finds reasons for hope.

## 

UN climate talks in Doha ended without much fanfare. At least the countries involved were able to kick the can down the road a bit, by extending the toothless and largely ineffective Kyoto Protocol to 2015. Meanwhile, global CO_2_ emissions set a new record high in 2012.

So are we doomed? This is the question people mostly ask when I speak publicly as a climate scientist. Americans seem to either believe that global warming is not happening, somehow, or that it has happened already. The first conclusion is demonstrably wrong, but I believe that the second conclusion is also unwarranted. Most of the carbon that we worry about, the carbon that will drive the truly apocalyptic climate changes projected for the end of the century, is still in the ground.

Consider that, if there were simply no more coal on the planet, the global warming problem would be a lot smaller. Coal is the dominant fossil fuel, comprising about 90% of the fossil carbon reserves. Although economics and extraction technology may extend the availability of oil and gas, to really toast the planet requires a burning a lot of coal.

Human progress would not grind to a halt if there were no more coal. We are simply too creative and innovative to return lamely to the days of human and animal muscle as our primary energy sources. If the coal suddenly ran out, the scramble for alternative energy would be an opportunity you would read about in the business section, not an existential threat to humanity.

The hardest part is making the decision. It is a case of “tragedy of the commons”, in which the costs of the decision to use fossil energy are not paid by the users, but are paid “externally” by others, especially people in the future and in the developing world. The natural inclination of a collection of selfish people in this situation is to over-exploit the common resource at the expense of all.

But humankind does seem to me to be evolving ethically. It’s not always obvious from one decade to the next, but over the centuries human ethical evolution seems clear. We have largely eliminated slavery as an institution, for example, not because it was financially expedient (in fact it was probably costly to the beneficiaries of that system to give it up), but because it was the ethical thing to do. We no longer crucify people, or hang them in the public square, or burn them alive.

I also feel that social systems, such as our energy infrastructure and its influence on our government, seem tippy and impossible to predict. No one foresaw the timing of the fall of the Berlin wall or the beginning of the Arab Spring. The repressive governments of the Eastern bloc and the Middle East seemed like they could last forever, and then suddenly everything changed.

The American public has not been convinced by the pronouncements of climate scientists, but 80% of Americans have experienced some form of extreme weather in the last five years, attributable in some measure to a human impact on climate. If the forecast is right, this is only the beginning. The weather is going to keep getting weirder, and the droughts deeper. One laudable goal of civilization is to protect people from unfair harm inflicted by others, and the changing weather will ultimately convince people that CO_2_ emission causes other people harm. Also, my sense is that (how to put this delicately?) the turnover of the human population over the decades will play a role in the evolution of public perception of the climate change issue.

But is it too late? Already Earth’s climate is changing, if anything more quickly than had been forecast. There is built-in inertia to the climate system, which means that even if atmospheric CO_2_ concentrations stopped rising, Earth’s temperature would continue to rise for centuries. The major ice sheets in Greenland and Antarctica have already started losing water (more quickly than anyone had really forecast), and the sea ice in the Arctic is collapsing (astonishingly quickly). Have we already passed a point of no return?

Components of Earth’s climate system can sometimes “flip” abruptly into alternate states, in some cases to get stuck there. The melting of the Arctic sea ice is clearly a state change, potentially affecting the circulation of the North Atlantic and the stability of the Greenland ice sheet. However, melting the Arctic sea ice is probably not an irreversible step, in that if Earth cooled down again, the sea ice would return [1]. Other transitions have been identified that would be irreversible, such as the melting of the great ice sheets in Greenland and Antarctica, and the release of carbon from melting permafrost and ocean methane hydrate pools. However, these are slow transitions, stretching into the centuries ahead, giving us some time. While the possibility of surprises must be acknowledged, there is no concrete reason to just give up all hope now.

The numbers are these. We have already released about 500 Gton C (1 Gton equals 10^15^ g) from fossil fuels and deforestation. This is about as much carbon as in all the trees in the world. By the end of the century, under business as usual and assuming no effort to avoid climate disruption, the total burn could be 2000 Gton C. Ultimately there are generally thought to be about 5000 Gton C in fossil fuels (mostly coal) [2].

However, the amount of carbon we can burn will probably be limited by the climate, rather than by the availability of fuel. If in the end humankind burns about 1000 Gton C (we’re halfway there), the temperature of the Earth is projected to rise by about 2°C [3,4]. Warming of 2°C would exceed anything that civilized, agricultural humanity has ever experienced, and warmer than Earth has been in millions of years. So this is not a “safety limit” so much as a benchmark to talk about, roughly marking a turning point in Earth’s climate. Beyond 2°C the projected impacts intensify, becoming different in kind as well as severity [5]. Rain forests and ice sheets begin to collapse, and the likelihood increases of abrupt surprises.

At current rates of emission, we would reach 1000 Gton C cumulative emission in a few decades, sooner if the current acceleration of emission continues. In practicality it would be hard to quit burning fossil fuels cold turkey, so we need to start sooner. A reasonable rate of decrease of say 3% cuts per year would result in an ultimate net carbon emission slug of about the 1000 Gton target. The longer we wait, the faster the drawdown would have to be, in order to come in under the 1000 Gton mark [2].

If things get too extreme, it is possible to extract CO_2_ from the atmosphere [6], like cleaning up the world’s biggest oil spill. The U.S. would get a big bill in this scenario, as we are responsible for about a quarter of the CO_2_ load so far. Also note that the oceans, helping us now by absorbing CO_2_, would give the CO_2_ back if we pulled the atmospheric concentration down. Economically, if scrubbing the atmosphere is our ultimate plan, it’s pretty stupid to continue releasing CO_2_ today. But if it comes down to saving the world, CO_2_ extraction from the atmosphere should be doable.

It’s clear that the cheapest and safest course would be to begin cutting global CO_2_ emissions a quickly as possible, as deeply as possible. It’s also clear that so far this isn’t happening. But in the long run I see reasons for hope. You may say that I’m a dreamer, but I’m not the only one. Imagine carbon neutral! We can do it if we try.
